# Kawasaki Disease in the neonate: case report and literature review

**DOI:** 10.1186/s12969-018-0263-8

**Published:** 2018-07-03

**Authors:** Fajer Altammar, Bianca Lang

**Affiliations:** 1 Department of Pediatrics, IWK Health Centre, Dalhousie University, 5980 University Ave, Halifax, NS B3K 6R8 Canada; 2 Division of Rheumatology, Department of Pediatrics, IWK Health Centre, Dalhousie University, 5980 University Ave, Halifax, NS B3K 6R8 Canada

**Keywords:** Kawasaki, Infant, Diagnosis, Newborn, Neonate

## Abstract

**Background:**

Kawasaki Disease (KD), the leading cause of acquired heart disease in children in the developed world, is extremely rare in neonates. We present a case of incomplete KD in a neonate and a review of the literature on neonatal KD.

**Case presentation:**

A previously healthy full term 15 day old Caucasian male with an unremarkable antenatal and perinatal history, presented on Day 2 of illness with fever, rash, irritability, and poor feeding. Examination revealed fever (39.6C), tachycardia, tachypnea, extreme irritability, and a generalized maculopapular rash, but was otherwise normal. His complete blood count, CRP and ESR were normal. Empiric intravenous antibiotics and acyclovir resulted in no improvement. On day 4, he had ongoing fever and developed recurrent apnea, required supplemental oxygen, and was transferred to the pediatric intensive care unit. On day 6, he developed bilateral non-purulent conjunctivitis, palmar erythema, bilateral non-pitting edema and erythema of his feet, and arthritis. His full septic work-up and viral studies were negative. On Day 7 he was treated with intravenous immunoglobulin, and over the next 48 h his symptoms including extremity edema resolved, he no longer required supplemental oxygen, and fever did not recur. On day 9 of illness he had marked thrombocytosis. Subsequently, he developed distal extremity desquamation. Repeated echocardiograms excluded the presence of coronary artery aneurysms (CAA).

**Conclusions:**

We believe this to be a rare case of incomplete KD in a neonate, in which timely IVIG administration led to resolution of the acute illness and may have prevented CAA. A comprehensive English-language medical literature review of KD presenting in the neonatal period revealed only fifteen case reports. Cases often presented with incomplete KD, and a number had atypical laboratory features including a normal CRP in the acute phase, similar to what was seen in our patient. This case and our literature review should increase awareness that KD can rarely occur in neonates, often presenting atypically. Recognizing KD in a neonate enables appropriate treatment that can result in resolution of symptoms and may decrease the risk of cardiac complications.

## Background

Kawasaki Disease (KD), a vasculitic syndrome of early childhood with a predilection for the coronary arteries, is now the leading cause of acquired heart disease in children in the developed world. The underlying etiology is still unknown, and there is no definitive diagnostic test. The diagnosis of complete KD is based on the presence of ≥ 5 days of fever and 4 or more of the principal clinical criteria including erythematous changes of the lips and oral mucosa, bilateral non-purulent conjunctivitis, a nonspecific rash, erythema and edema of the feet and hands or periungual desquamation (subacute phase), and cervical lymphadenopathy (≥1.5 cm). Incomplete KD is diagnosed in patients with unexplained prolonged fever and less than 4 criteria, if they have typical echocardiographic findings of KD or typical laboratory features [1]. Prompt treatment of KD patients with IVIG has been shown to reduce the incidence of coronary artery aneurysms from 25% to less than 5% [1–3].

The age of onset of KD is usually between 6 months and 5 years of age. It is much less common under 6 months of age and rare in children under 3 months of age. It is especially rare in the neonatal period, with only a few neonatal cases ever reported in the literature. Even in Japan, where KD is much more prevalent than elsewhere in the world, very few neonates with KD have been identified. Data on 105,755 KD patients from the first 12 Japanese nationwide surveys of KD (1970–1992), identified 1768 KD cases (1.67%) 90 days old or younger. Of these, only six cases were of age 30 days old or younger, with the youngest being 26 days old [4]. In a review of the Japanese nationwide surveys of KD from 2001 to 2012, 23 neonates were identified, representing 0.02% of KD patients overall [5]. We present a case of suspected incomplete KD in a neonate and a review of the literature on neonatal KD with the aim to increase awareness that KD can occur in the first month of life and may present with atypical features.

## Case presentation

**Fig. 1:**
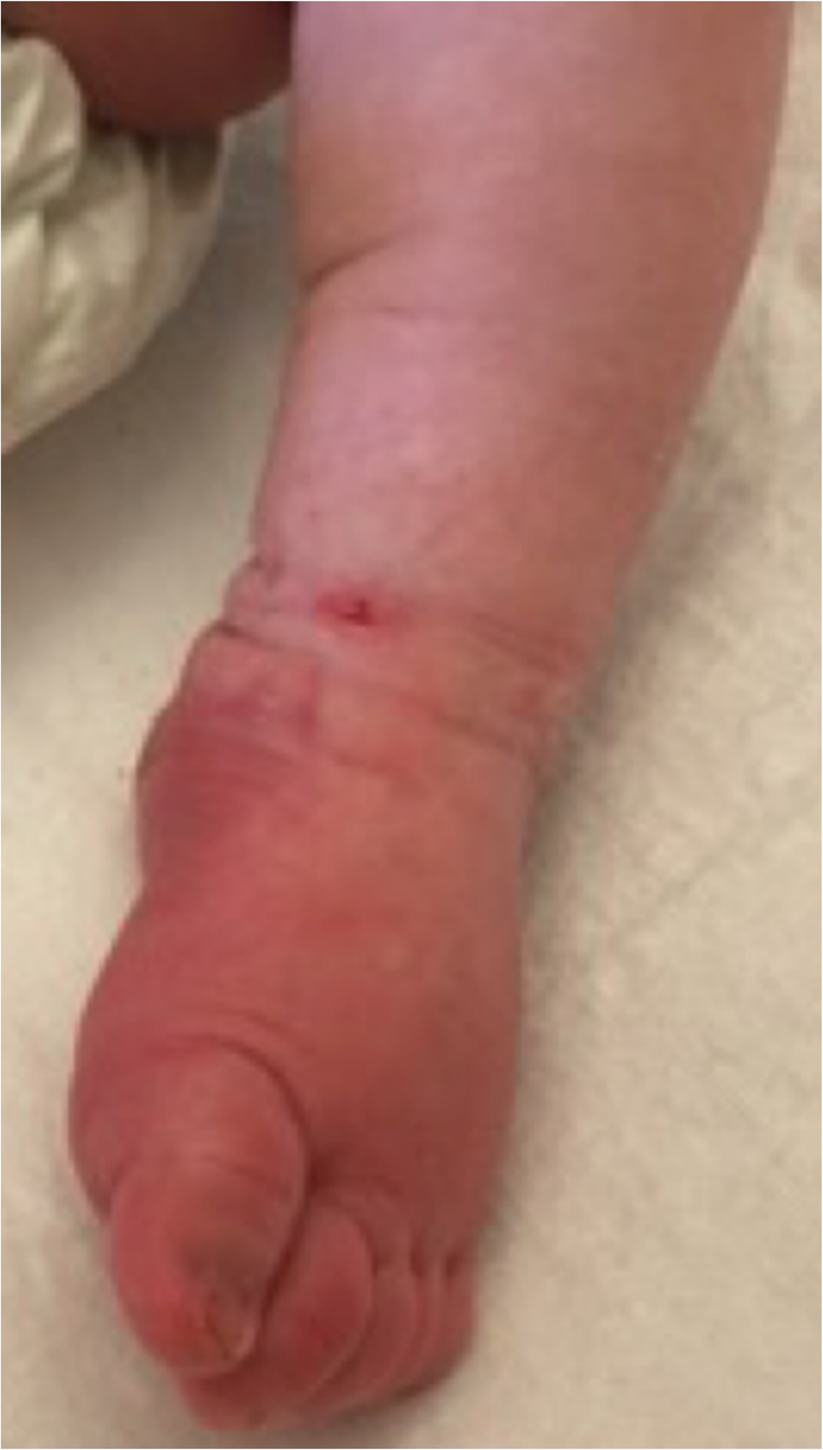
Photograph demonstrating extremity changes including marked non-pitting edema and erythema of foot

A previously healthy full term 15 day old Caucasian male with an unremarkable antenatal, perinatal, and family history presented on Day 2 of illness with a 24 h history of poor feeding, irritability, fever, and rash. Examination revealed fever (39.6 C), tachycardia (HR 180–210), tachypnea (RR 68), extreme irritability, acrocyanosis, and a generalized maculopapular rash, but otherwise was normal. Admission blood work revealed a normal complete blood count, slightly elevated serum transaminase levels, mild hypoalbuminemia (30 g/L), and a normal c-reactive protein (CRP) of 2.6 mg/L. Empiric treatment with intravenous ampicillin, cefotaxime, and acyclovir was given for presumed neonatal sepsis and possible encephalitis. On day 4 of illness, in addition to ongoing fever, he developed recurrent apnea and required supplemental oxygen and transfer to the tertiary care hospital pediatric intensive care unit. His blood work showed mild neutropenia, elevation of serum transaminase levels (alanine transaminase 86 U/L, aspartate transaminase 220 U/L), a normal ESR (1 mm/hr) and hypoalbuminemia. An infectious etiology was considered unlikely given negative bacterial cultures from blood, cerebral spinal fluid, and urine, and negative viral studies for herpes simplex virus 1 and 2, Respiratory Syncytial Virus, Influenza A and B, cytomegalovirus, Epstein Barr virus, rubella, adenovirus, and rotavirus. The urine and blood cultures were collected before the start of antibiotics. His chest radiograph was normal. On day 6 of illness, he developed bilateral non-purulent conjunctivitis, palmar erythema, striking bilateral non-pitting edema and erythema of his feet, and erythema and swelling of several proximal interphalangeal joints (Fig. 1). He continued to require supplemental oxygen for suspected pneumonitis. On Day 7 of illness, after 5 days of fever and meeting 3 out of 5 criteria for KD, with no other obvious diagnosis, he was given IVIG (2 g/kg) for suspected incomplete KD. Over the next 48 h, his symptoms including extremity edema resolved, he no longer required supplemental oxygen, and fever did not recur. His CRP remained normal throughout his illness. Laboratory values improved and his platelet count progressively increased from 246,000 on Day 7 to 852,000 on Day 13 of illness. In the subacute phase, he was treated with low dose aspirin. He had persistent thrombocytosis followed by typical extremity desquamation. Echocardiograms on weeks 1, 2, and 6, and at 12 months were normal with no carditis or coronary artery abnormalities.

## Discussion and conclusions

We report a rare case of incomplete KD in a neonate, where timely IVIG administration resulted in clinical improvement and may have prevented cardiac complications. Our patient had fever documented for 5 days and 3 additional principal clinical features including rash, bilateral non-purulent conjunctivitis, and very typical and pronounced non-pitting edema and erythema of his feet, as well as palmar erythema, and desquamation of the feet in the subacute phase. Other clinical features included extreme irritability, arthritis, apnea, and respiratory distress. Because of the rarity of KD in the neonatal period, a bacterial or viral etiology was suspected on admission, and the diagnosis of KD was not initially considered. However, after he had failed to improve with antibiotic and antiviral therapy, there was dramatic improvement of clinical features following IVIG. Although our patient did not have echocardiographic abnormalities to confirm a diagnosis of KD, he did have 3 typical laboratory findings including elevated ALT and AST levels, hypoalbuminemia, and marked thrombocytosis starting on day 9 of illness. Our literature review encompassed a thorough search of the electronic databases of PubMed and Embase for KD presenting in the neonatal period. The key terms used in the search included newborns, neonates, week-old, weeks old baby, babies, and Kawasaki or mucocutaneous lymph node syndrome. Only English articles were reviewed. The references of relevant publications were also reviewed to identify further cases that were not included in the databases. The neonatal period was defined as patients from birth to 28 days of age.

Our literature search revealed only a few reported cases of suspected neonatal KD worldwide (15 cases) (Table 1), confirming the rarity of this disorder, and consistent with the very few cases identified in Japan through nationwide surveys [4–15].

**Table 1:** Literature Review: Summary of the Case Reports of Neonatal Kawasaki Disease (0–28 days of age)

Pt	Ref	Age at onset^b^ (days)	Sex	Fever duration (days)	Rash	Oral changes	Extremity changes^d^	Red eyes	Cervical adenitis	CAA ^c^	Other	CRP (high/ WNL)	IVIG res-ponse	CA outcome (last F/U)
1	4	20	F	10	+	+	+	+	–	+	DIC	high	+	WNL (8mos)
2	6	10	M	5	+	+	+	+	–	+	Apnea, seizures	WNL	+	CAA (2.5 yrs)
3	7	8	F	9	+	+	+	+	–	+	MR/AR	high	+	WNL (6 wks)
4	8	16	F	13	+	+	+	+	–	+	MR/TR	high	+	N/A
5	9	20	F	N/A	+	+	+	–	–	+	MR/AR	high	+	CAA (8 wks)
6	10	8	M	> 9	+	–	+	–	–	+	Myocardial ischemia^a^	high	+	CAA (9 wks)
7	5	22	F	4	+	–	+	–	–	–	–	high	+	WNL (3 mos)
8	11	18	M	6	+	+	+	–	–	–	Pneumonitis	WNL	+	WNL (6 wks)
9	11	16	M	7	+	+	+	–	–	–	Cough, diarrhea	high	+	WNL (11 yrs)
10	12	21	F	4	+	+	+	–	–	–	Abdominal pain vomiting	WNL	+	WNL (6 wks)
11	12	14	F	3	+	+	+	–	–	–	Hyperemia	WNL	+	WNL (6 mos)
12	12	16	M	4	+	+	+	–	–	–	Hyperemia	WNL	+	WNL
13	13	5	M	1	–	–	–	–	–	+	CHF, MR/AR	high	+	WNL(12mos)
14	14	1	M	0	–	–	–	–	–	+	DIC, pericardial effusion	N/A	N/A	WNL(12mos)
15	15	1	F	0	–	–	–	–	–	+	MI, CA vasculitis	N/A	N/A	death (day1)
16	PR	15	M	5	+	–	+	+	–	–	Apnea pneumonitis	WNL	+	WNL(12mos)

*CAA* coronary artery abnormality, *CRP* C-reactive protein, *IVIG* intravenous immunoglobulin, *DIC* disseminated intravascular coagulation, *CHF* congestive heart failure, *AR* aortic regurgitation, *TR* tricuspid regurgitation, *MI* myocardial infarction, *PR* present report, *N/A* not available, *MR* mitral regurgitation, *WNL* within normal limits

^a^ Patient 6 underwent successful coronary thrombolysis

^b^ All patients were full term with the exception of patient #14 who was 35.5 weeks

^c^ Patients 2, 4, 5, 6, and 15 had CA aneurysms; patients 1, 2, 3, 13 and 14 had CA dilatations

^d^ Patients 1, 2, 3, 4, 5, 8, 9, 10, 11, 12 and 16 had distal edema; patients 1, 2, 3, 5, 6, 7, 8, 11 and 16 had desquamation

The infrequent occurrence of neonatal KD is consistent with the current theory of an infectious trigger for KD and the protection that most very young infants have through passive immunity transferred from their mother. The demographic and clinical features of the babies with suspected KD identified in our literature review are presented in Table 1. In all the presented cases including ours, most common infectious etiologies were excluded and all patients were empirically treated for sepsis. Four patients had complete KD, all with documented CAA. Of the 12 patients with fewer than 4 of 5 KD criteria, 6 infants, including our case, had fever with 2 or 3 criteria, and either CAA, or typical desquamation or thrombocytosis in the sub-acute phase, as well as resolution of symptoms following IVIG therapy. Three patients presented with 3–4 days of fever and 3 criteria, including striking extremity edema and erythema, and had rapid resolution of all symptoms with IVIG treatment at 3–4 days, despite not meeting KD criteria [12]. Three patients with suspected neonatal KD presented within the first 1–5 days of life with CAA suggestive of KD without prolonged fever or other principal criteria. While it is not possible to confirm their diagnosis, they may represent the spectrum of neonatal KD. Our case and literature review illustrate that KD in neonates commonly presents with incomplete features (75%). Table 2 summarizes the frequency of the clinical and laboratory manifestations of KD in our neonatal cases. None of the cases reviewed met the criteria for cervical lymphadenopathy, and few developed conjunctivitis. Fever, rash, and extremity changes were reported in most infants, oral changes in close to two thirds, and desquamation during the subacute phase was also common*.* Our findings are consistent with the high rate of incomplete KD reported in neonates identified in the Japanese nationwide surveys between 2001 and 2012, where only 8 of 23 infants had complete KD [5]. Our findings are also consistent with the high frequency of incomplete KD reported in infants less than 6 months of age who are beyond the neonatal period [16–20]. Moreover, the clinical manifestations in young infants can be short-lived and may not all manifest at any given time. Patients with incomplete KD who are less than 6 months of age, especially those lacking eye or oral mucosal changes appear to be particularly at risk for delays in diagnosis [21]. The absence of cervical lymphadenopathy in patients reported with neonatal KD supports the conclusions of Lee and Manlhiot that this criterion is not a sensitive indicator of KD in infants under 6 months of age, and suggests that this may be an important factor contributing to the lower likelihood of young infants meeting KD criteria [19, 20]. Our case report and literature review also demonstrate that a significant number of neonates with KD display unusual laboratory manifestations, including a normal CRP in the acute phase in 6/14 (43%), and thrombocytopenia in the acute phase of the illness in 4/12 (27%). In our case, the presence of a normal CRP on days 2 and 4 of his illness led to the early assumption that the diagnosis of KD was unlikely. Elevated inflammatory markers are reported in the vast majority of older infants with KD, and presence of a normal CRP generally suggests an alternative diagnosis [1, 22].

**Table 2:** Frequency of key clinical and laboratory features of 16 cases of NKD

Clinical and Laboratory Features	Frequency	(%)
Fever (any duration)	14/16	88
Duration of fever ≥ 5 days^a^	8/15	53
Rash	13/16	81
Extremity changes	13/16	81
Oral changes	10/16	63
Conjunctivitis	5/16	31
Cervical lymphadenopathy	0/16	0
Cardiac complications	9/16	56
Normal CRP^b^	6/14	43

^a^ Duration of fever not reported in 1 case. ^b^ CRP not reported in 2 cases

Several studies have suggested that infants under the age of 6 months not only present more commonly with incomplete KD, but are also at higher risk for coronary artery abnormalities and death [16, 20, 23–25]. According to Hangai et al., from nationwide Japanese surveys, the risk of coronary abnormalities was 17% among the 23 neonates diagnosed with Kawasaki disease [5]. Among the neonatal cases we reviewed, the frequency of cardiac complications was even higher (56%). This may in part be due to the fact that the diagnosis of KD in three cases was made based on the presence of typical cardiac abnormalities alone. Krapf et al. described a neonate who presented with fatal myocardial infarction without any clinical features of KD, who at autopsy, was found to have an acute/subacute necrotizing coronary vasculitis typical of KD [15]. In a more recent case report by Parashar et al., bilateral coronary artery dilatations were detected on the first day of life in the absence of other KD features [14]. Bolz et al. described a baby who presented during his first week of life in congestive heart failure who was found to have dilatation of his left coronary arteries without other KD features [13]. In the latter two cases, the coronary dilatation resolved; in the second case this occurred after IVIG therapy. As KD is a clinical diagnosis, it is not possible to confirm the diagnosis of KD in these three infants. However, they did not have another known cause of CA dilatation, such as asphyxia, sepsis, congenital cardiac anomaly, other recognized systemic vasculitis or systemic juvenile arthritis, and they did not have prolonged fever, which itself may result in CA dilatation, although this is usually transient, with Z scores less than 2.5 [1]. The fatal neonatal case reported by Krapf et al. is strikingly similar to cases of infantile polyarteritis nodosa (IPN), a uniformly fatal vasculitis of infancy with a predilection for CAs. A review of 20 cases of IPN from North America published four years before Dr. Kawasaki reported the criteria for KD in Japan described the clinical features seen in many, but not all, infants with IPN, including fever, rash, conjunctivitis, abnormal urinary sediment, and cardiomegaly [26]. A decade after the description of KD, Landing and Larson reviewed the clinical and pathologic features of 20 patients with IPN and compared these with two fatal cases of KD, and concluded that the two conditions were clinically and pathologically indistinguishable [27]. Following the worldwide recognition of KD, reports of IPN essentially disappeared [28]. The neonate reported by Krapf et al. had CA pathology typical of IPN and KD, supporting the concept that IPN is the severe end of the spectrum of incomplete KD. The absence of fever was recognized years ago in some infants with IPN, and has recently been reported in three patients suspected to have “afebrile KD” [29, 30]. Two of these patients from Japan had bilateral conjunctivitis, erythema at their BCG sites, and CA Z scores ≥ 8.0, while one 3-month infant from North America had no clinical features of KD other than CAA (Z score ≥ 6.0). These cases, as well as the NKD cases we present, challenge us to keep a high index of suspicion for KD, especially in young infants, as the criteria Dr. Kawasaki developed were not designed to identify infants at risk of CAA, but rather, to make a diagnosis of what he believed at the time was a benign, self-limited febrile illness.

To help clinicians recognize incomplete cases of KD and reduce the delay in diagnosis that predisposes to CAA, particularly in high-risk infants under the age of 6 months, the American Heart Association (AHA) 2017 guidelines provide recommendations for the evaluation of suspected incomplete KD [1]. According to the AHA diagnostic algorithm, in children with 5 or more days of fever and 2 or 3 compatible clinical criteria, or in infants with fever for 7 or more days without another explanation, the presence of an elevated CRP or ESR warrants further laboratory and/or echocardiographic testing for KD [1]. In the subgroup of patients whose CRP is less than 3.0 mg/dl and/or ESR < 40 mm/hour, serial clinical and laboratory re-evaluation is warranted only if fever persists, and an echocardiogram is recommended if typical peeling develops. While this approach may successfully identify older infants with KD, the AHA diagnostic algorithm may result in a delay in diagnosis and treatment of neonates with KD, as our literature review and case report illustrate that incomplete features are common and a normal CRP may occur in the acute illness. Ideally, a diagnostic test for KD will be found, otherwise more inclusive diagnostic criteria may be needed for this age group so that the diagnosis of KD is not delayed. Pannaraj et al. surveyed general pediatricians and pediatric infectious disease physicians in San Diego using a questionnaire to understand physician practices in diagnosing Kawasaki disease and showed that 71 of 124 (57.3%) general pediatricians and 86 of 324 (26.5%) pediatric infectious disease specialists did not consider the diagnosis of KD in children younger than 6 months of age in their differential diagnosis [31]. Increasing awareness of KD and its unusual presentation in young infants may be the most important intervention for these patients.

In conclusion, this case, in addition to the cases presented in our review, illustrate the importance of considering the diagnosis of KD in the first month of life, as appropriate treatment can result in resolution of symptoms and a decreased risk of cardiac complications. It is important to recognize that neonatal KD, while rare, often poses a diagnostic challenge. Incomplete KD is common, and unusual features such as a normal CRP in the acute phase can be seen in a significant number of cases. It is essential to maintain a high index of suspicion for such a diagnosis in febrile neonates who fail to respond to antibiotics, while also recognizing the limitations of applying the AHA guidelines for diagnosis in this age group.

## Abbreviations

ALT: Alanine transaminase
AST: Aspartate transaminase
CAA: Coronary Artery Aneurysms
CRP: C-reactive protein
ESR: Erythrocyte sedimentation rate
IPN: Infantile polyarteritis nodosa
IVIG: Intravenous immunoglobulin
KD: Kawasaki Disease
NKD: Neonatal Kawasaki Disease

## References

[1] McCrindle BW, Rowley AH, Newburger JW, Burns JC, Bolger AF, Gewitz M, American Heart Association Rheumatic Fever, Endocarditis, and Kawasaki Disease Committee of the Council on Cardiovascular Disease in the Young; Council on Cardiovascular and Stroke Nursing; Council on Cardiovascular Surgery and Anesthesia; and Council on Epidemiology and Prevention (2017). Diagnosis, treatment, and long-term Management of Kawasaki Disease: a scientific statement for health professionals from the American Heart Association. Circulation.

[2] Furusho K, Nakano H, Shinomiya K (1984). High-dose intravenous gammaglobulin for Kawasaki disease. Lancet.

[3] Newburger JW, Takahashi M, Burns JC (1986). Treatment of Kawasaki syndrome with intravenous gamma globulin. N Engl J Med.

[4] Tsuchida S, Yamanaka T, Tsuchida R, Nakamura Y, Yashiro M, Yanagawa H (1996). Epidemiology of infant Kawasaki disease with a report of the youngest neonatal case ever reported in Japan. Acta Paediatr.

[5] Hangai M, Kubota Y, Kagawa J, Yashiro M, Uehara R, Nakamura Y (2014). Neonatal Kawasaki disease: case report and data from nationwide survey in Japan. Eur J Pediatr.

[6] Stanley TV, Grimwood K. Classical Kawasaki disease in a neonate. Arch Dis Child Fetal Neonatal Ed. 2002; 10.1136/fn.86.2.F135. 10.1136/fn.86.2.F135 PMC1721383 11882560

[7] Nakagawa N, Yoshida M, Narahara K, Kunitomi T (2009). Kawasaki disease in an 8-day-old neonate. Pediatr Cardiol.

[8] Thapa R, Pramanik S, Dhar S, Kundu R (2007). Neonatal Kawasaki disease with multiple coronary aneurysms and thrombocytopenia. Pediatr Dermatol.

[9] Bhatt M, Anil SR, Sivakumar K, Kumar K (2004). Neonatal Kawasaki disease. Indian J Pediatr.

[10] Karia VR, Hescock GC, Gedalia A, Ross-Ascuitto N (2010). Successful emergent coronary thrombolysis in a neonate with Kawasaki's disease. Pediatr Cardiol.

[11] Mitchell S, Francis J, Burgner D (2011). Kawasaki disease in the neonatal period. J Pediatr Infect Dis.

[12] Nasir A, Al Tatari H, Hamdan MA (2012). Very high serum ferritin levels in three newborns with Kawasaki-like illness. Paediatr Child Health.

[13] Bolz D, Arbenz U, Fanconi S, Bauersfeld U (1998). Myocarditis and coronary dilatation in the 1st week of life: neonatal incomplete Kawasaki disease?. Eur J Pediatr.

[14] Parashar R, Lysecki PJ, Mondal T (2013). Diffuse coronary artery dilatation in a neonate: a case report. J Neonatal Perinatal Med.

[15] Krapf R, Zimmerman A, Stocker F (1981). Lethal vasculitic of coronary arteries in a neonate and two infants: possible neonatal variant of the MLNS/IPN complex?. Helv Paediatr Acta.

[16] Burns AC, Wiggins JW, Toews WH, Newburger JW, Leung DYM, Wilson H (1986). Clinical spectrum of Kawasaki disease in infants younger than 6 months of age. J Pediatr.

[17] Rosenfeld EA, Corydon KE, Shulman ST (1995). Kawasaki disease in infants less than one year of age. J Pediatr.

[18] Joffe A, Kabani A, Jadavji T (1995). Atypical and complicated Kawasaki disease in infants – do we need criteria?. West J Med.

[19] Lee KY, Hong JH, Han JW, Lee JS, Lee JS, Burgner D (2006). Features of Kawasaki disease at the extremes of age. J Paediatr Child Heath.

[20] Manlhiot C, Yeung R, Clarizi N, Chaha N, McCrindle B (2009). Kawasaki disease at the extremes of the age spectrum. Pediatrics.

[21] Minich LL, Sleeper LA, Atz AM, McCrindle BW, Lu M, Colan SD, et al. Pediatric heart network investigators. Delayed diagnosis of Kawasaki disease: what are the risk factors? Pediatrics. 2007; 10.1542/peds.2007-0815. 10.1542/peds.2007-0815 18025079

[22] Koyanagi H, Yanagawa H, Nakamura Y, Yashiro M (1997). Serum C-reactive protein levels in patients with Kawasaki disease: from the results of nation-wide surveys of Kawasaki disease in Japan. Acta Paediatr.

[23] Chang FY, Hwang B, Chen SJ (2006). Characteristics of Kawasaki disease in infants younger than six months of age. Pediatr Infect Dis J.

[24] Singh S, Agarwal S, Bhattad S, Gupta A, Suri D, Rawat A (2016). Kawasaki disease in infants below 6 months: a clinical conundrum?. Int J Rheum Dis.

[25] Salgado AP, Ashouri N, Berry EK, Sun X, Jain S, Burns JC, et al. High risk of coronary artery aneurysms in infants younger than 6 months of age with Kawasaki disease. J Pediatr. 2017; 10.1016/j.jpeds.2017.03.025. 10.1016/j.jpeds.2017.03.025 PMC5529235 28408126

[26] Roberts FB, Fetterman GH (1963). Polyarteritis Nodosa in infancy. J Pediatr.

[27] Landing BH, Larson EJ (1977). Are infantile Periarteritis Nodosa with coronary artery involvement and fatal Mucocutaneous lymph node syndrome the same? Comparison of 20 patients from North America with patients from Hawaii and Japan. Pediatrics.

[28] Burns JC (2018). History of the worldwide emergence of Kawasaki disease. Int J Rheum Dis.

[29] Yoshino A, Tanaka R, Takano T, Oishi T (2017). Afebrile Kawasaki disease with coronary artery dilatation. Pediatr Int.

[30] Pinches H, Dobbins K, Cantrell S, May J, Lopreiato J. Asymptomatic Kawasaki disease in a 3-month-old infant. Pediatrics. 2016:e20153936. 10.1542/peds.2015-3936. 10.1542/peds.2015-3936 27371760

[31] Pannaraj PS, Turner CL, Bastian JF, Burns JC (2004). Failure to diagnose Kawasaki disease at the extremes of the pediatric age range. Pediatr Infect Dis J.

